# Lamivudine and Zidovudine-Loaded Nanostructures: Green Chemistry Preparation for Pediatric Oral Administration

**DOI:** 10.3390/nano13040770

**Published:** 2023-02-18

**Authors:** Marina D. V. Guedes, Morgana S. Marques, Simone J. Berlitz, Murilo H. M. Facure, Daniel S. Correa, Clarice Steffens, Renata V. Contri, Irene C. Külkamp-Guerreiro

**Affiliations:** 1 Programa de Pós-Graduação em Ciências Farmacêuticas, Universidade Federal do Rio Grande do Sul, Porto Alegre 90610-000, RS, Brazil; 2 Programa de Pós-Graduação em Nanotecnologia Farmacêutica, Universidade Federal do Rio Grande do Sul, Porto Alegre 35400-000, RS, Brazil; 3 Laboratório Nacional de Nanotecnologia para o Agronegócio (LNNA), Embrapa Instrumentação, São Carlos 70770-901, SP, Brazil; 4 Programa de Pós-Graduação em Química (PPGQ), Universidade Federal de São Carlos, São Carlos 66075-110, SP, Brazil; 5 Programa de Pós-Graduação em Engenharia de Alimentos, Universidade Regional Integrada do Alto Uruguai e Missões, Erechim 99709-910, RS, Brazil; 6 Programa de Pós-Graduação em Farmacologia e Terapêutica, Universidade Federal do Rio Grande do Sul, Porto Alegre 90050-170, RS, Brazil

**Keywords:** children, cubosomes, green chemistry, nanoemulsion, oral administration

## Abstract

Here, we report on the development of lipid-based nanostructures containing zidovudine (1 mg/mL) and lamivudine (0.5 mg/mL) for oral administration in the pediatric population, eliminating the use of organic solvents, which is in accordance with green chemistry principles. The formulations were obtained by ultrasonication using monoolein (MN) or phytantriol (PN), which presented narrow size distributions with similar mean particle sizes (~150 nm) determined by laser diffraction. The zeta potential and the pH values of the formulations were around −4.0 mV and 6.0, respectively. MN presented a slightly higher incorporation rate compared to PN. Nanoemulsions were obtained when using monoolein, while cubosomes were obtained when using phytantriol, as confirmed by Small-Angle X-ray Scattering. The formulations enabled drug release control and protection against acid degradation. The drug incorporation was effective and the analyses using an electronic tongue indicated a difference in palatability between the nanotechnological samples in comparison with the drug solutions. In conclusion, PN was considered to have the strongest potential as a novel oral formulation for pediatric HIV treatment.

## 1. Introduction

HIV/AIDS (Acquired Immunodeficiency Syndrome) disease continues to be a major global public health issue, especially in the child population. Vertical transmission and sexual infection are the main causes of the disease in this population, which is more vulnerable and presents lower viral suppression levels than adults [1]. Infants and young children living with HIV have an elevated risk of poor outcomes. Up to 52% of children born with HIV die before the age of 2 in the absence of any intervention [2].

Despite the high number of children living with AIDS, there are only a few liquid pharmaceutical formulations for pediatric use. Among them, the combination of lamivudine (3TC) and zidovudine (AZT) is the first line of nucleoside reverse transcriptase inhibitors’ (NRTI) backbone for pediatric treatment [3]. However, this treatment is administered in tablets with a fixed dosage, and no liquid formulation is available on the market combining these two drugs, which are currently difficult to administer to young children. It is possible to attribute the dearth of pediatric formulations to several factors. First, the child population is a small part of the general population, which does not motivate the pharmaceutical industry to put in the resources necessary to produce pediatric products. Additionally, the technology necessary to make the medications more palatable, the selection of components, and the difficulty in making them easy to take must be considered [4].

Thus, the development of novel child-friendly formulations is a key step to be reached. In this context, the use of nanotechnology for the development of pediatric medicines is the aim of this study. This technology could provide stable liquid formulations, with taste-masking properties and easy dose adjustment capability [4]. A progressive and controlled release of the drug can be achieved with a more convenient administration of zidovudine and lamivudine in the same formulation [5].

In recent years, lipids such monoolein and phytantriol have been used in the development of nanocarriers due to their great potential as drug delivery systems [6]. Monoolein is a nontoxic and biodegradable material, used in cosmetics, foods, and pharmaceutical products, and is classified as GRAS (generally recognized as safe) and also included in the FDA Inactive Ingredients Guide [7,8]. In pharmaceutical products, monoolein can be used as emulsifier, solubilizer, absorption enhancer, and in oral drug delivery systems, such as those proposed in this work [7]. Phytantriol is also nontoxic and biocompatible and is widely used in the cosmetic industry [9]. Phytantriol was considered nontoxic in acute oral testing and not genotoxic while having low skin penetration [10]. Moreover, phytantriol could improve drug bioavailability [11].

Here, we developed lipid-based nanostructures containing zidovudine and lamivudine without the addition of organic solvents, resulting in a more sustainable formulation, aligned with the principles of green chemistry by minimizing or eliminating the use and generation of hazardous substances [12]. This nanosystem was developed through the technique of multiple nanoincorporation using monoolein and phytantriol as the main nanostructure ingredients and originated a liquid pharmaceutical formulation containing antiretroviral drugs proposed for the treatment of pediatric HIV/AIDS disease.

## 2. Materials and Methods

### 2.1. Materials

Lamivudine and zidovudine were kindly donated by Farmanguinhos (Rio de Janeiro, RJ, Brazil). Monoolein was purchased from Sigma-Aldrich (Burlington, MA, US) (40% TLC degree of purity). Phytantriol was obtained from Alianza (São Paulo, Brazil). Poloxamer 407 was acquired from BASF (São Paulo, SP, Brazil), and mucin from Lab Confiança (Diadema, SP, Brazil). Sodium chloride, 37% hydrochloric acid, and sodium hydroxide were purchased from Neon (São Paulo, Brazil). Methanol (HPLC grade) was obtained from Sigma-Aldrich (Burlington, MA, US). All other solvents were of analytical grade.

### 2.2. Preparation of Lipid-Based Nanostructures

Two types of lipid-based nanostructures containing zidovudine and lamivudine were developed, varying the type of polar lipid, as described in Table 1. There was no use of organic solvents during the nanostructure production, which is in accordance with the green chemistry principles [13].

The drugs were dispersed with magnetic stirring in monoolein or phytantriol (70 °C). This homogeneous lipid phase was mixed with a previously prepared 407 poloxamer aqueous solution for 10 min at 40 °C. A tip sonicator (QR 350 Watt Ultrasonic—Ultronic^®^ Eco-Sonics probe type) was used to reduce the particle size (99% power for 15 and 30 min, for formulations MN and PN, respectively) maintaining the formulation in a water bath at 40 °C (top-down approach). Monoolein-based nanostructures and phytantriol-based nanostructures without drugs were also prepared for evaluating the specificity of the drug quantification method. All formulations were prepared in triplicate. The 2:1 (*w/w*) polar lipid:poloxamer 407 ratio was chosen based on previous published results [14]. The formulations were stored in amber flasks until use. 

### 2.3. Characterization of Lipid-Based Nanostructures

#### 2.3.1. Particle Size and Size Distributions

Particle mean size and particle size distribution were determined by laser diffraction (Mastersizer^®^ 2000, Malvern Panalytical, Malvern, UK). In the laser diffraction technique, the samples were directly added into the wet dispersion unit containing distilled water. Particle mean size was expressed as the volume-weighted mean diameter (D[4,3]), and particle size distribution was expressed as Span, calculated on a volume (v) basis using Equation (1):

Span = d(0.9) − d(0.1)/d(0.5)(1)

where d(0.1), d(0.5), and d(0.9) are the particle diameters at the 10th, 50th, and 90th percentiles of the particle size distribution curve, respectively.

#### 2.3.2. Zeta Potential

Zeta potential values were determined by electrophoretic mobility (Zetasizer Nano ZS^®^, Malvern Panalytical, Malvern, UK) after samples were diluted (1:1000 *v/v*) in a 1 mM NaCl aqueous solution previously filtered (0.45 µm, Kasvi, São José dos Pinhais, PR, Brazil).

#### 2.3.3. pH Measurements

The pH of samples was determined at 25 °C without prior dilution using a calibrated (pH 4.0 and 7.0) potentiometer (DM-22, Digimed, São Paulo, SP, Brazil).

#### 2.3.4. Lamivudine and Zidovudine Content and Incorporation Efficiency

The AZT and 3TC content of monoolein-based nanostructures and phytantriol-based nanostructures were quantified using a high-performance liquid chromatography method with UV detection (HPLC-UV) (Shimadzu, Kyoto, Japan) based on a previously described method (Beck, 2007). A C18 column (Waters Atlantis T3, 4.6 mm × 150 mm, 5 µm, Milford, MA, US) with a C18 guard column (Phenomenex, Torrance, CA, US), citrate buffer/methanol (50:50 *v/v*) mobile phase at pH 6.5, flow rate of 0.8 mL min^−1^, and λ = 270 nm were applied. Prior to HPLC-UV analysis, samples were diluted in methanol/water (70:30 *v/v*) and filtered (0.45 µm, Chromafil, Düren, Germany).The HPLC-UV method was previously validated by investigating specificity, using the nanostructures without drugs as matrix; linearity in the range of 21 to 39 µg mL^−1^ for 3TC and 42 to 78 µg mL^−1^ for AZT, intraday precision by analyzing six samples at the same concentration; and interday precision, by analyzing samples with different concentrations on different days.

AZT and 3TC incorporation into MN and PN nanostructures were evaluated by ultrafiltration-centrifugation of samples in a filter device (Microcon, Millipore 10,000 Da, Burlington, MA, US) at 4500 rpm (Laborzentrifugen 2K15, Sigma, Osterode am Harz, Germany) for 10 min. The ultrafiltrates were directly analyzed by HPLC-UV with dilution (1:10 *v/v*) in methanol/water (70:30 *v/v*). Drug loading efficiency (IE) was expressed as a relative percentage, calculated by the ratio of the difference between the total drug content and the drug content of the ultrafiltrate to the total drug content, Equation (2):

IE (%) = (drug content − free drug/drug content) × 100(2)

#### 2.3.5. Differential Scanning Calorimetry (DSC)

For the formulations MN and PN, each individual component and the physical mixture of the components in the same proportions used in formulations were evaluated using a DSC, which has been previously calibrated. The DSC analysis was carried out using DSC Q2000 (TA Instruments, New Castle, DE, US) equipment; N_2_ gas at a flow rate of 50 mL/min. The samples were placed in platinum pans, which were sealed and scanned at a rate of 10 °C/min over a temperature range of 0–90 °C (two heating cycles and one cooling cycle). An empty pan was used as reference.

#### 2.3.6. Small Angle X-ray Scattering Measurements (SAXS)

SAXS experiments were performed in Xenocs^®^ Nano-InXider equipment, which operates with Dectris^®^ Pilatus3 detector and CuKα X-ray source (λ = 0.154 nm), being calibrated with silver behenate standard (Centro de Nanociências e Nanotecnologia, CNANO/UFRGS, Porto Alegre, RS, Brazil). The formulations were transferred to one-aperture thin glass capillaries (Charles Supper^®^, Natick, MA, US), which were sealed and placed in a liquid sample holder. Consecutive 60 s measurements were obtained at room temperature over 3 h for each sample in the ‘medium resolution’ mode. The same procedure was performed for a capillary containing water, whose signal was subtracted from the sample signal. The reduction of 2D images to 1D curves was performed using Foxtrot^®^ version 3.2.7 (SOLEIL, Saint-Aubin, France). The data were fitted to mathematical models using the SASFit^®^ software.

#### 2.3.7. Scanning Electron Microscopy (SEM)

The morphology of the formulations was evaluated by scanning electron microscopy (SEM), using a Zeiss equipment (model EVO LS25, Berlin, Germany). The samples were coated with a mixture of gold (80%) and palladium (20%) in a vacuum chamber.

### 2.4. Storage Stability Studies

Monoolein-based and phytantriol-based nanosystems were stored in glass flasks and kept under refrigeration (4 ± 0.5 °C) or at room temperature (25 ± 0.5 °C) for up to 30 days. At the end of the experiment, the formulations were evaluated in terms of particle size, particle size distribution, pH, and AZT/3TC content.

### 2.5. In Vitro Drug Release Profiles

The in vitro release of AZT and 3TC from nanoformulations was investigated by the dialysis bag method [15,16] comparing the nanostructures with the drug solution in purified water at the same concentration (FS).

The closed cellulose dialysis bags (25 mm, MWCO 12 kDa, Sigma–Aldrich, Burlington, MA, USA) containing 2 mL of samples were immersed in 80 mL of release medium (purified water) and kept in a water bath (37 ± 0.5 °C) under magnetic stirring. During a period of 8 h, 1 mL of release medium was withdrawn at predetermined intervals, filtered (0.45 μm, Chromafil, Germany), and measured by the previously mentioned HPLC–UV method. This aliquot was replaced by an equal volume of fresh release medium (37 ± 0.5 °C). The experiment was performed in triplicate.

### 2.6. Drug Degradation in Simulated Acid Medium

Monoolein-based and phytantriol-based nanostructures, as well as drug aqueous solutions (4 mL), were placed in contact with simulated acid medium (4 mL, USP specifications—simulated gastric fluid (SGF) pH 1.2 containing sodium chloride, 37% hydrochloric acid, and purified water) at 37 °C under magnetic stirring. After 15, 30, and 60 min, samples were collected and analyzed by the previously mentioned HPLC–UV method [17].

### 2.7. Mucoadhesiveness

Mucin solution was prepared in water at 0.25 % (*w/v*) concentration under magnetic stirring at room temperature. Aliquots of nanostructures were mixed with mucin solution (1:1) at 37 °C for 2 h. The resulting solutions were evaluated regarding mucoadhesion index (MI) [18]. The MI were calculated according to Equation (3):

MI = Dh2/Dh0(3)

where Dh0 and Dh2 are the average particle sizes before and after contact with mucin, respectively. The experiment was carried out in triplicate.

### 2.8. In Vitro Taste Masking Evaluation

Nanostructured thin films have been widely used to develop sensing units of electronic tongues, once this can usually increase sensitivity and performance of the e-tongues. In this case, four sensing units were obtained using poly (allylamine hydrochloride) (PAH), polyethyleneimine (PEI), sulfonated polystyrene (PSS), and graphene oxide (GO). The films were obtained using the layer-by-layer (LbL) technique, alternating the depositions of the cationic polymers (PAH and PEI) and the anionic polymer (PSS) and GO, thus obtaining the films PAH/PPS, PAH/GO, PEI/PSS, and PEI/GO. The material solutions for the LbL assembling were prepared using HCl (pH 3.0). Four bilayers were used in each film. The films were deposited onto gold interdigitated electrodes (IDE). Each IDE was composed of 50 pairs of fingers, with a width and gap between fingers of 10 μm, obtained through the photolithography technique at the microfabrication laboratory (LMF/LNNano-LNLS, Campinas-Brazil). The films were obtained by soaking the electrode in the material solution for 15 min for each layer. After each layer deposition, the electrode was washed in an HCl solution (pH 3.0).

The lipid-based nanostructures as well as lamivudine and zidovudine solutions were measured, separately, by using an e-tongue. All tested samples contained lamivudine and zidovudine at concentrations of 0.5 μg/mL and 1.0 μg/mL, respectively. To analyze the palatability of the samples, in addition to the analyses carried out with the drug solutions; solutions representing the basic tastes were also analyzed: sucrose (sweet), HCl (sour), NaCl (salty), quinine (bitter), and glutamate (umami). All solutions were prepared with ultrapure water at a concentration of 1 mmol L^−1^.

Impedance spectroscopy measurements were performed using a Solartron impedance analyzer (1260A). All analyses were performed by recording electrical resistance and capacitance values as a function of frequency, which was varied from 1 MHz to 10 Hz, using a constant applied voltage of 50 mV.

### 2.9. Statistical Analysis

Results were analyzed for statistical significance by one-way analysis of variance (ANOVA) followed by Tukey’s test at a significance level of 0.05 using GraphPad Prism version 5.0. The data collected by the e-tongue were treated using the PEx-Sensors software and Origin version 8.5.

## 3. Results and Discussion

### 3.1. Preparation and Characterization of the Lipid-Based Nanostructures

The formulations were prepared using phytantriol or monoolein as polar lipids, poloxamer 407 as a stabilizer, and water as the external phase. No organic solvent was used for the nanoformulation preparation. Therefore, costs are reduced along with avoiding potentially toxic solvents, which brings more safety regarding the target audience [19].

Laser diffraction was used as a preliminary analysis to determine the average diameter and the presence of particles in the micrometer range as well as the span value, which indicates the particle size distribution pattern. The most suitable ultrasonication time for MN was 15 min, while for PN, 30 min was needed to achieve a similar nanometer size distribution (Table 2). The results revealed particles in the nanometer size range by volume, with unimodal size distributions. The formulations (n = 3 batches) presented white homogeneous macroscopic aspects (not shown), without any precipitation.

The zeta potential was −4.38 mV and −3.09 mV for MN and PN, respectively. These values of zeta potential are in accordance with the presence of poloxamer 407 in the components of the nanocarrier, which enables the stabilization of nanocarriers due to steric properties [20,21,22]. Average pH was 5.8 ± 0.1 for MN and 6.2 ± 0.1 for PN.

The HPLC-UV method proved to be specific for the drugs and linear between 21–39 µg/mL (r = 0.994) for lamivudine and 42–78 µg/mL (r = 0.994) for zidovudine. The lamivudine content was 96.4 ± 4.3% and 96.6 ± 2.8 for MN and PN, respectively, with respect to a theoretical value of 0.5 mg/mL. The incorporation rate (%) of lamivudine was 67.2 ± 2.5 (MN) and 59.2 ± 8.1 (PN). Zidovudine content was 101.3 ± 5.7% and 109.6 ± 4.6 for MN and PN, respectively, with respect to a theoretical value of 1.0 mg/mL. The zidovudine incorporation rate (%) was 75.2 ± 3.9 (MN) and 72.6 ± 9.2 (PN). MN presented a higher incorporation rate compared to PN, for both drugs. In addition, comparison of the drugs showed that zidovudine was incorporated at a higher extent, probably due to its higher lipophilicity.

The results of incorporation efficiency (IE) for all formulations are similar to those described in the literature for nanostructures prepared using different methods and compositions, but with the same drugs. Kumar et al. [23] found about 65% and 71% IE for these drugs, with a lower rate for lamivudine.

Astolf et al. [24] found low IE values by incorporating a hydrophilic drug (logP –0.66). They found an incorporation rate for the drug 5-fluorouracil of 25.7 to 45.3%. Thus, the method and the components selected for the preparation of MN and PN nanostructures in the present study were suitable and very efficient for the incorporation of these hydrophilic drugs, when compared to other studies that had lower incorporation rates, varying according to the amphiphilic character of the lipids used.

Gavini and co-workers [25] obtained liposomes with lamivudine and zidovudine in order to obtain a more stable system with an improvement in the therapeutic index for both drugs. Additionally, Sankar and co-workers [26] obtained polymeric nanostructures co-encapsulating the two same drugs. Both authors used lamivudine and zidovudine as the drugs to be incorporated in nanostructures; however, Sankar and collaborators (2012) [26] produced polymeric nanostructures and further evaluated their distribution systems in the body, while Gavini and colleagues [25] developed liposomes. Both studies did not intend to produce green or pediatric formulations.

**Figure 1:**
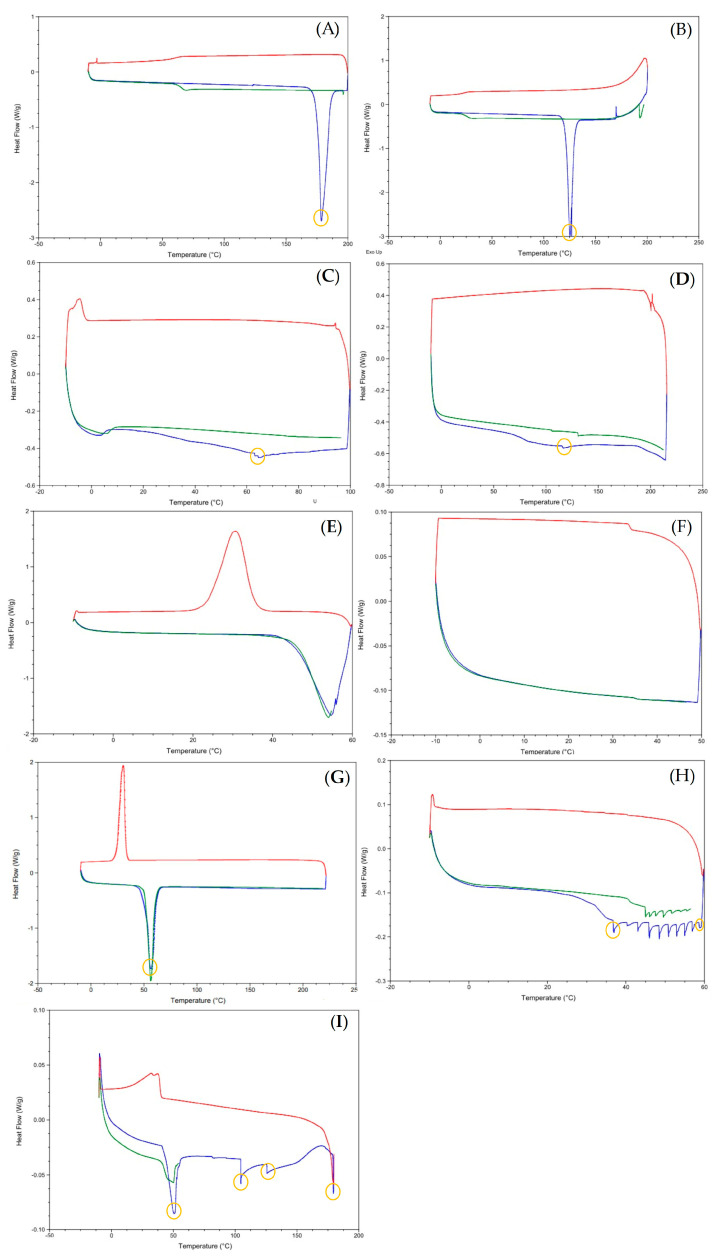
Thermograms of 3TC (**A**), AZT (**B**), monoolein (**C**), phytantriol (**D**), monoolein-based nanostructures—MN (**E**), phytantriol-based nanostructures—PN (**F**), poloxamer (**G**), physical mixture of MN formulation ingredients (**H**), and physical mixture of PN formulation ingredients (**I**). Lines: 10°C/50 min (green), 10 °C/100 min (blue), 10 °C/200 min (red).

DSC thermograms are shown in Figure 1, with the characteristic peaks highlighted by yellow circles. DSC thermograms of pure drugs (3TC and AZT), monoolein, phytantriol, and poloxamer presented broad endothermic peaks between 125–135 °C (Figure 1A), 175–180 °C (Figure 1B), 65–70 °C (Figure 1C), 110–125 °C (Figure 1D), and 50–60 °C (Figure 1G), respectively. These values indicate the melting points of the samples. The physical mixture of monoolein and the other components in the same proportion used to prepare the MN showed a broad endothermic peak between 35 °C and 60 °C (Figure 1H). On the other hand, the physical mixture of phytantriol and the other components (including water) in the same proportion used to prepare the PN showed an endothermic peak between 50 and 55 °C, between 100 and 110 °C, at 125 °C, and between 175 and 180 °C (Figure 1I).

It can be seen that the characteristic peaks of the drugs (Figure 1A,B) change with nanoformulations (Figure 1E,F), mainly in the PN formulation (Figure 1F), in which the peaks disappear, evidencing an interaction between drugs and nanoformulations [27,28]. When only physical mixing occurs, the peaks continue to appear, showing that it is necessary to follow the top-down preparation process to promote the interaction between the drug and the other components in a molecular way.

In order to determine the phase structure of the nanostructures in each formulation, SAXS measurements were performed. Diffraction peaks indicated the existence of a liquid crystalline phase for formulations prepared with phytantriol, with or without the drug. Each peak is associated with a family of crystallographic planes. The analysis of the relative distances of the peaks (qn/q1) in lyotropic liquid crystals allows the phase structure to be determined [29]. The curves of these samples show diffraction peaks with relative positions of q in √2: √3: √4: √6: √8: √9, which are compatible with the reverse bicontinuous cubic structure of the Pn3m spatial group. The Miller indices (hkl) associated with these peaks are (110), (111), (200), (211), (220), and (221), respectively [30,31]. Considering these results, the phytantriol-based formulations can be considered cubosomes, which is expected for nanoparticles prepared using this polar lipid [9].

The diameter of the water channels (d_w_) in a Pn3m reverse bicontinuous cubic phase (d_w_) can be calculated from its estimated radius (r_w_) (Equation (4)) as described by Briggs and collaborators [32]:

r_w_ for Pn3m = 0.391a-*l* (4)

where *l* is the length of the monolayer, and a is the lattice parameter. This value corresponds to 1.42 nm for phytantriol [33]. The lattice parameter a for cubic phases can be obtained from the inverse of the slope of dhkl-1as a function of $\sqrt{h^{2}+k^{2}+l^{2}}$, where dhkl = 2π/qn. The values of a for the phytantriol-based formulations were both 6.7 nm, regardless of the presence of the drug. Consequently, the calculated rw value corresponded to 1.2 nm for both samples. It is suggested, therefore, that the drug did not cause expansion or contraction of the aqueous channels.

In addition to the peaks associated with the reverse bicontinuous cubic phase with the spatial group Pn3m, an additional peak was observed at q = 0.147 Ǻ. The lack of other additional peaks related to this in the range of q analyzed with an adequate signal-to-noise ratio makes it impossible to elucidate the phase structure to which it refers. Additional studies involving transmission electron microscopy could assist in its elucidation.

The formulations prepared with technical grade monoolein did not show diffraction peaks, indicating that they do not have an internal liquid crystalline structure, as expected. In this respect, the samples differ from systems composed of high purity monoolein previously described in the literature [34,35]. This result can be explained by the composition of technical grade monoolein, which contains 40% (*w/w*) of 1-oleoyl-rac-glycerol and the other 60% is a mixture of diglyceride and triglyceride. The use of pure monoolein can form different liquid crystalline phases such as a lamellar phase, two bicontinuous cubic phases, and a reverse hexagonal phase [36]. Hence, using a low grade monoolein can hinder the formation of these phases as indicated by the SAXS evaluation. Since diglyceride and triglyceride are fatty acids, they can be in the lipid-phase of the nanoemulsion [37,38,39]. As it does not have a structure factor, we sought to describe the internal structure of the nanostructures based on their form factor using the BroadPeak model (Equation (5)):

*l*(q) = I_0_/1 + (|q-q_0_|ξ)^m^ (5)

In this model, d is the characteristic distance between scattering inhomogeneities, which is related to the position of the broad peak in the SAXS curve as q = 2π/d. The other parameters are the correlation length ξ, a power law exponent m, and the scattered light intensity I_0_ when q = 0.

The *d-spacing* value corresponded to 4 nm, regardless of the presence of the drug. The lipid core does not show an ordered internal structure, suggesting that the monoolein-based formulation can be described as nanoemulsion. It should be noted that an attempt was made to adjust to the Teubner–Strey model, which describes a sponge phase or bicontinuous microemulsion [40], but the model in question was not able to adequately describe the experimental data. In this sense, the interior of the nanostructures is not likely to contain aqueous channels typical of this type of structure.

**Figure 2:**
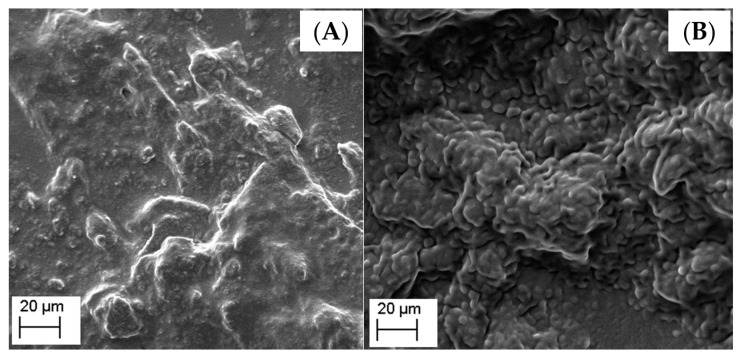
Scanning electron microscopy images of cubosomes (**A**) and nanoemulsion (**B**).

Figure 2 shows the SEM images obtained for both developed formulations. It was observed that both formulations had a nanometric size. The PN formulation, in which it is responsible for forming cubosomes (Figure 2A), has a more misshapen morphology, not being possible to clearly identify the nanostructures. The formed cubosome morphology was heterogeneously distributed clusters, with a particle aggregation tendency, which may be caused by the drying of the cubosome suspensions. The cubosomes could have had their structure influenced by the loss of water from the formulation, during the preparation of the samples. Therefore, other techniques such as cryo-TEM or AFM could be more suitably applied to such type of nanostructures [41,42,43,44]. On the other hand, the MN morphology (Figure 2B) showed particles with a spherical shape and uniform distribution, which is in agreement with nanoemulsion morphology [45,46,47,48,49]. The SEM analysis showed that the developed nanostructures are different as confirmed by SAXs analysis.

### 3.2. Storage Stability Studies

**Figure 3:**
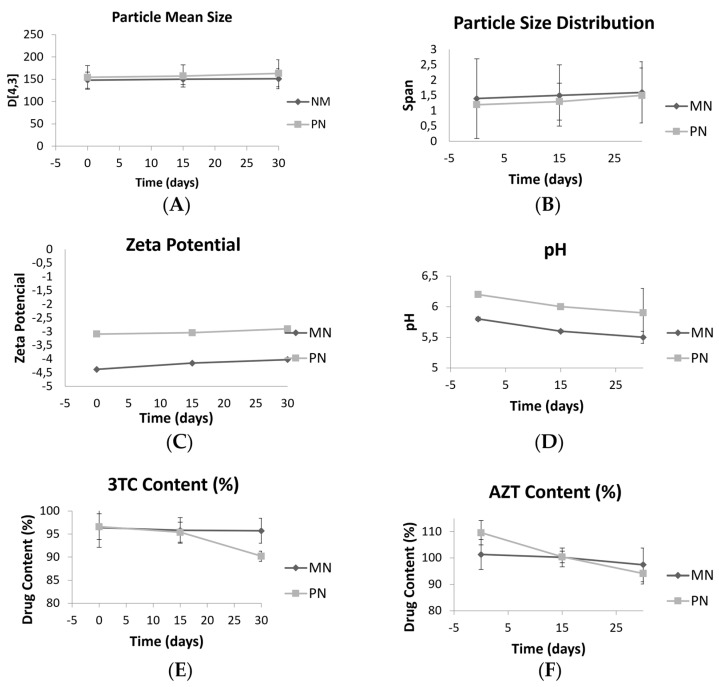
Size D[4,3](V) (**A**), span(V) (**B**), zeta potential (**C**), pH (**D**), 3TC content (**E**), and AZT content (**F**) of formulations at times 0, 15, and 30 days under storage at room temperature. MN: monoolein-based nanostructures. PN: phytantriol-based nanostructures.

Storage stability studies are essential to guarantee the quality of formulations for biomedical applications. Monoolein-based nanostructures and phytantriol-based nanostructures were stored at room temperature and under refrigeration for 30 days. The formulations were evaluated in terms of particle mean size, particle size distribution, zeta potential, pH, and 3TC/ATZ content (Figure 3). After one day under storage, the samples under refrigeration were physically not stable as phase separation occurred. Therefore, no more evaluations were performed, and they were discarded.

However, after 30 days of storage at room temperature, the particle size of all samples remained stable (Figure 3A), as well as the unimodal size distribution, with no statistical changes over time. The SPAN values (Figure 3B) showed no significant increase after 30 days, and the zeta potential values (Figure 3C) did not show remarkable variations. Concerning the pH (Figure 3D), both formulations showed a slight reduction after 30 days of preparation (*p* < 0.05). The pH became more acidic and the zeta potential increased. 3TC content (Figure 3E) did not change during 30 days for MN, but for PN, a reduction from 96.6 to 90.2% was observed (*p* < 0.05). AZT content (Figure 3F) reduced for both nanostructures (from 101.3 to 97.4% for MN and from 109.6 to 94.1% for PN), but the values were still within acceptable specifications (110 to 90%, according to the Brazilian Pharmacopeia) [50].

### 3.3. In Vitro Drug Release Profiles

**Figure 4:**
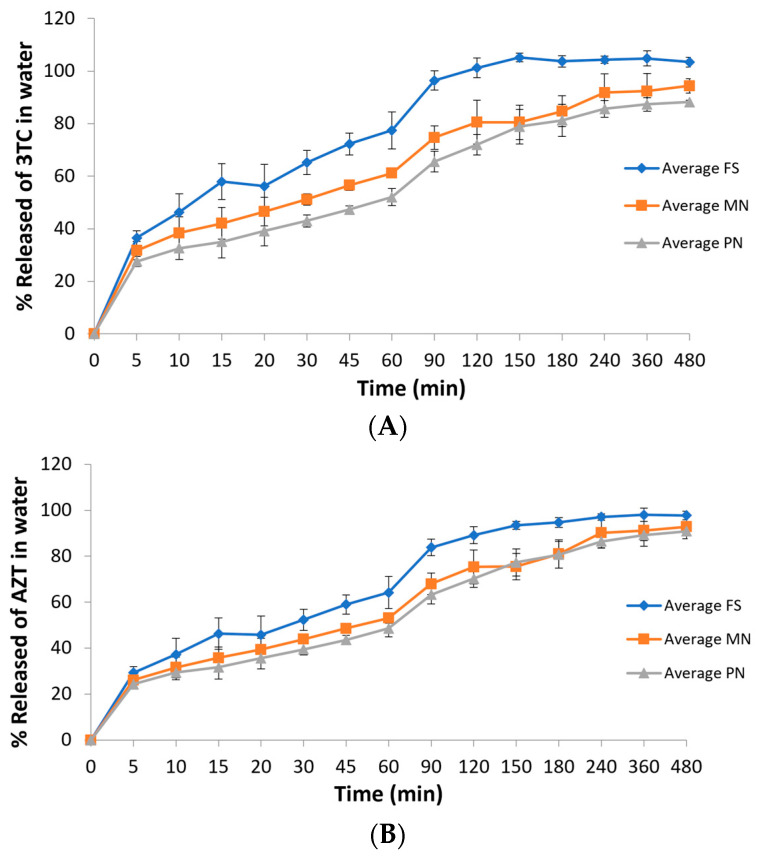
In vitro 3TC (**A**) and AZT (**B**) release profiles in water from FS, MN, and PN formulations. FS: free drug solution; MN: monoolein-based nanostructures; PN: phytantriol-based nanostructures.

Water was selected as the release medium since sink condition can be achieved with no drug degradation. The percentages of 3TC and AZT release as a function of time are shown in Figure 4. Regarding 3TC, it can be observed that drug release reached 100% after 120 min for the drug in solution (FS). While FS reached a 100% release in 120 min, MN reached around 80% and PN reached 72% at the same time. Both MN and PN did not reach 100% of drug release during the time of the experiment (480 min), indicating a slower drug release when compared to the drug in solution. Considering AZT, FS reached around 98% of drug release at the end of the experiment (480 min). However, a slightly lower percentage was observed for both nanocarriers at the same time. The nanocarrier MN reached around 93% of AZT release at 480 min while PN achieved a lower percentage of around 91% at the same time. These data indicate that both nanoformulations were able to increase drug release time, especially for 3TC, when compared to the drugs in solution. Since PN and MN can be described as cubosome and nanoemulsion, respectively, a burst release can be expected and depends on the nature of the surfactants [51].

Regarding the area under the curve of the graphs, a statistical difference can be seen between the free drug solution (FS) and both nanostructures (PN and MN), but between the two types of nanostructures, there is no significant difference (Table 3). Such data can indicate that the nanocarriers appear to increase drug release time when compared to the drugs in solution. This difference is interesting since both drugs are hydrophilic and a quick diffusion from the carriers to the aqueous medium is expected [52,53].

### 3.4. Drug Degradation in Acid Medium

The degradation percentages of the drugs in acid medium after 1 h of contact are shown in Table 4. The average gastric emptying time in children is 150 min [54]. This gastric emptying process varies greatly from person to person, due to the specific physiological characteristics of each individual, in addition to the presence or absence of food. The time period of 1 h was chosen as a pattern in that process. Both nanostructures decreased the percentage of drug degradation in acid medium, compared to the drug solution. PN presented higher efficacy when protecting the drugs from degradation, when compared to MN formulation.

These results indicate the greater ability of PN formulations to protect the antiretroviral drugs from degradation in acidic medium compared to MN. This is probably due to the different morphological structures of nanostructures. According to Avachat and Parpani (2015) [55], monoolein degrades in acid medium. These lipids have an ester bond and are therefore susceptible to gastrointestinal tract (TGI) degradation with consequent loss of liquid crystal structure observed for pure monoolein-based formulations. This effect was previously observed with in vitro digestion models in monoolein-based cubosomes. These authors tested phytantriol, an undigested monoolein analogue with a phase behavior very similar to monoolein, which resulted in the preservation of the liquid crystalline structure in the TGI using phytantriol and sustained the release of efavirenz over a period of 12 h improving its bioavailability. The drug efavirenz is lipophilic; in this sense, the behavior of lipids in protecting hydrophilic drugs in the gastric environment was not known, showing the importance of this result.

### 3.5. Mucoadhesiveness

The mucoadhesive properties of the nanostructures were assessed by the ability to interact with porcine gastric mucin (type II). Mucin, one of the main components of the mucus layer, is a glycoprotein responsible for its viscoelastic gel properties that is directly involved in adhesion phenomena. Human and animal mucin present similar chemical nature and morphology [56,57]. Besides being already extensively described in studies of particle and mucin interaction, the porcine gastric mucin was suggested by Teubl et al. as a model of human mucin [56].

The mucin solution had a micrometer size, while the nanostructures had average sizes of 82 ± 12 nm and 147 ± 3 nm for the MN and PN formulations, respectively, before contact with the mucin. All nanostructures showed unimodal distribution profiles with PDI values below 0.2 (0.13 ± 0.05 for the MN formulation and 0.09 ± 0.004 for the PN formulation). After contact with mucin, the formulations showed bimodal distribution profiles with a main peak at the nanometer scale similar to that shown for the nanostructure before contact, and other small peaks in the micrometer range, probably referring to mucin. The mean sizes for MN and PN after contact with mucin were 82 ± 1 nm and 165 ± 4 nm, respectively. These values of average diameter resulted in MI values of 1.11 for PN. The formulation MN, on the other hand, did not demonstrate mucoadhesiveness (MI = 0.99). An increase in particle size indicates the nanoformulation aggregated with the mucin particles, thus having an affinity for them [18,58]. The results indicate that the mucin is partially adsorbed on the surface of the nanostructures, while a remaining amount was kept free in the solution, with respect to the PN formulation.

The ability to interact with the mucin presented by the phytantriol-based nanostructures demonstrates its mucoadhesive properties. The mechanism for the adhesion of nanostructures is probably a dynamic balance between electrostatic interactions and other mechanisms, such as diffusion and physical entanglement [57,59].

Considering the relatively short gastrointestinal time, the phenomenon of mucoadhesion is very important, as it leads to a significant improvement in the location of drugs for sustained oral delivery [60,61].

### 3.6. In Vitro Taste Masking Evaluation

The analysis of basic tastes can be performed using an impedimetric electronic tongue (e-tongue), which has shown the capability to overcome the human threshold [62]. Detection, differentiation, and recognition of the basic tastes using an impedimetric e-tongue have been widely reported [63]. For this purpose, conductive polymers have been used to obtain the sensing units of the e-tongue. The materials are used in the form of thin films, using the Langmuir–Blodgett (LB) or self-assembly layer-by-layer (LbL) techniques, and have shown excellent results in flavor identification [64,65,66,67,68].

**Figure 5:**
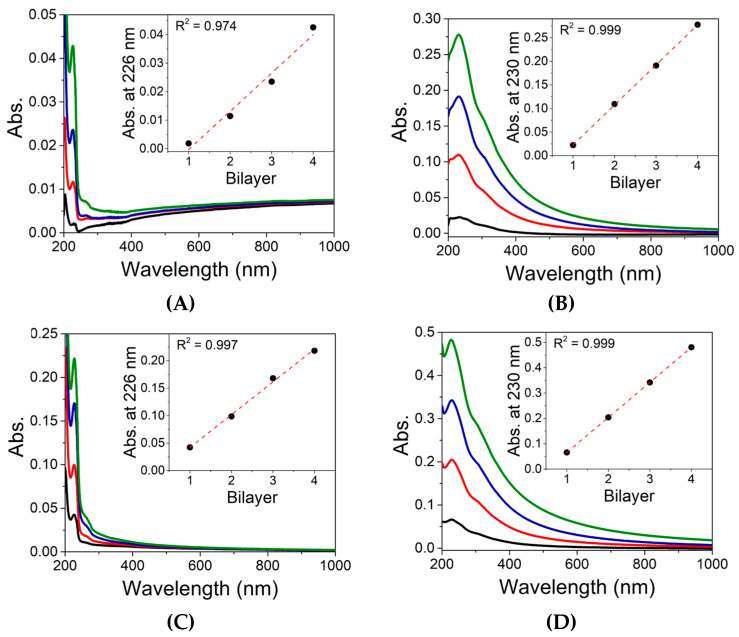
UV–vis spectra obtained after the deposition of each bilayer for (**A**) PAH/PPS, (**B**) PAH/GO, (**C**) PEI/PSS, and (**D**) PEI/GO LbL films.

In our case, we employed the LbL technique, and the film growth monitoring was performed through UV–vis spectroscopy. Figure 5 shows the spectra obtained from the films after the deposition of each bilayer in glass slides. The insets show a linear relationship between the absorbance value and the bilayer deposition for all the films obtained. The linear curves were obtained using the absorption value measured at 226 nm and 230 nm for the films containing PSS and GO, respectively. The results indicate a uniform growth of the films after the deposition of each bilayer and the successful production of the modified sensing units.

The obtained sensing units were used in an impedimetric e-tongue for the analysis of drugs and nanoformulations drugs.

Such results suggest that the free drug solution can have a more bitter taste than the nanoformulations. The results also show that the e-tongue was capable of identifying and distinguishing the basic tastes and all the solutions containing the drugs.

**Figure 6:**
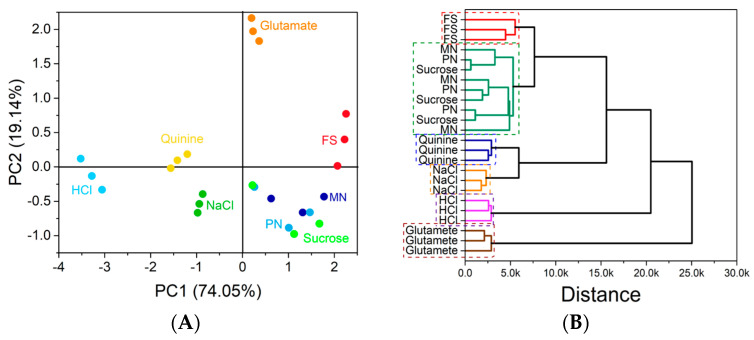
PCA graph obtained with the resistance data collected at the frequency of 1000 Hz for the drug solutions and the solutions used to mimic the basic tastes (**A**). Dendrogram obtained from the cluster analysis performed with electrical resistance data collected at the frequency of 1000 Hz (**B**). FS: free drug solution (lamivudine + zidovudine); MN: monoolein-based nanostructures; PN: phytantriol-based nanostructures.

The electrical resistance data collected at a fixed frequency (1000 Hz) were treated using the principal component analysis (PCA) technique. Figure 6A shows the obtained PCA graph.

It is possible to notice that there is a distinction between the samples of the nanoformulations (PN and MN) and the drug solution sample (FS). While the PN and the MN samples are located in the same quadrant, i.e., presenting positive PC1 and negative PC2 values, the FS samples are located in a region with positive PC2 values. From the PCA, it is possible to note again that the e-tongue was effective in recognizing the different tastes and in identifying each sample. In this analysis, however, the e-tongue was not able to clearly distinguish the nanoformulation samples (PN and MN) from the sucrose solution used. This fact can be explained by the chemical similarity between the lipids used in the formulations to the sucrose structure. The PCA plot represents 93.19% of all the variance collected by the e-tongue system.

A clustering analysis was also performed with the data used in the PCA. Figure 6B shows the resulting dendrogram. By cluster analysis, it is possible to notice that once again the e-tongue was able to differentiate nanoformulation samples from free drug solutions. The e-tongue was again able to identify all basic tastes, grouping together the nanoformulations containing the drugs and the sucrose solution.

## 4. Conclusions

We have successfully developed nanoformulations containing lamivudine and zidovudine through a green chemistry process, which have shown nanometric size with adequate size distribution. The pH was found suitable for the administration via oral route. Based on the parameters analyzed, it can be concluded that the association of lamivudine and zidovudine were possible in the nanocarriers proposed. The nanoformulations were prepared without hazardous solvents and present applicability for children (pleasant taste), in addition to obtaining a modified hydrophilic drug release form that is a difficult advantage to achieve. Therefore, it was possible to achieve the initial objective of sustainable development of a liquid pharmaceutical formulation for multiple nanoincorporation of lamivudine and zidovudine for pediatric oral use. The formulation that achieved the most favorable results was PN due to the gastric resistance of the polar lipid used as a component of the structure, combined with a hydrophilic surface that efficiently entrapped the drugs. The perspective of this study includes biological analysis of toxicity and in vivo efficacy evaluation.

## Figures and Tables

**Table 1:** Composition of the lipid-based nanostructures containing antiretroviral drugs. (Amount described for a final volume of 20 mL).

Formulation	Monoolein	Phytantriol	Poloxamer 407	Zidovudine	Lamivudine
MN	2 g	-	1 g	20 mg	10 mg
PN	-	2 g	1 g	20 mg	10 mg

MN: monoolein-based nanostructures. PN: phytantriol-based nanostructures.

**Table 2:** Volume-based (v) particle size (D[4,3]) and particle size distribution (Span) for monoolein-based and phytantriol-based nanostructures, measured by laser diffraction (mean ± SD).

	Monoolein-Based Nanostructures		Phytantriol-Based Nanostructures	
Ultrasonication Time (min)	D[4,3] (v) (nm)	Span (v)	D[4,3] (v)	Span (v)
5	288 ± 62	3.0 ± 0.6	1253 ± 389	8.8 ± 0.5
10	191 ± 32	1.9 ± 0.4	563 ± 15	7.6 ± 1.1
15	148 ±18	1.4 ± 1.3	437 ± 42	6.4 ± 0.8
20	ND	ND	259 ± 53	3.1 ± 1.7
25	ND	ND	171 ± 35	1.4 ± 0.4
30	ND	ND	154 ± 27	1.2 ± 0.1

ND: not determined.

**Table 3:** Area under the curve for 8 h release profiles of drugs 3TC and AZT.

Formulation	Drug Analyzed	AUC (In Vitro Release Profile)
FS	Lamivudine	46,539 ± 1411 ^a^
MN	Lamivudine	39,590 ± 2548 ^b^
PN	Lamivudine	36,743 ± 1380 ^b^
FS	Zidovudine	42,437 ± 692 ^a^
MN	Zidovudine	38,016 ± 2545 ^b^
PN	Zidovudine	36,763 ± 1297 ^b^

Different letters in the same column indicate significant differences between values (*p* < 0.05) by the Tukey test. FS: free solution or drug solution; MN: monoolein-based nanostructures; PN: phytantriol-based nanostructures.

**Table 4:** 3TC and AZT degradation for 1 h in simulated acidic medium.

Formulation	Drug analyzed	Drug Degradation in Acid Medium (%)
FS	Lamivudine	18.2 ± 2.7^a^
MN	Lamivudine	15.0 ± 6.8^a^
PN	Lamivudine	6.3 ± 5.4^b^
FS	Zidovudine	18.0 ± 3.1^a^
MN	Zidovudine	13.6 ± 8.7^a^
PN	Zidovudine	4.9 ± 6.3 ^b^

Different letters in the same column indicate significant differences between values (*p* < 0.05). FS: free solution or drug solution; MN: monoolein-based nanostructures; PN: phytantriol-based nanostructures.

## Data Availability

The data presented in this study are available on request.
